# Robust, Long-Term Video EEG Monitoring in a Porcine Model of Post-Traumatic Epilepsy

**DOI:** 10.1523/ENEURO.0025-22.2022

**Published:** 2022-07-08

**Authors:** Luis Martinez-Ramirez, Andrea Slate, George D. Price, Ann-Christine Duhaime, Kevin J. Staley, Beth A. Costine-Bartell

**Affiliations:** 1 Department of Neurosurgery, Massachusetts General Hospital, Boston, Massachusetts 02114; 2 Center for Comparative Medicine, Massachusetts General Hospital, Boston, Massachusetts 02114; 3 Department of Neurosurgery, Harvard Medical School, Boston, Massachusetts 02115; 4 Department of Neurology, Massachusetts, Boston, Massachusetts 02114; 5 Department of Neurology, Harvard Medical School, Boston Massachusetts, 02115

**Keywords:** electroencephalogram, gyrencephalic, interictal spikes, post-traumatic epilepsy, semiology, videotelemetry

## Abstract

To date, post-traumatic epilepsy (PTE) research in large-animal models has been limited. Recent advances in neocortical microscopy have made possible new insights into neocortical PTE. However, it is very difficult to engender convincing neocortical PTE in rodents. Thus, large-animal models that develop neocortical PTE may provide useful insights that also can be more comparable to human patients. Because gyrencephalic species have prolonged latent periods, long-term video EEG recording is required. Here, we report a fully subcutaneous EEG implant with synchronized video in freely ambulatory swine for up to 13 months during epileptogenesis following bilateral cortical impact injuries or sham surgery The advantages of this system include the availability of a commercially available system that is simple to install, a low failure rate after surgery for EEG implantation, radiotelemetry that enables continuous monitoring of freely ambulating animals, excellent synchronization to video to EEG, and a robust signal-to-noise ratio. The disadvantages of this system in this species and age are the accretion of skull bone, which entirely embedded a subset of skull screws and EEG electrodes, and the inability to rearrange the EEG electrode array. These disadvantages may be overcome by splicing a subdural electrode strip to the electrode leads so that skull growth is less likely to interfere with long-term signal capture and by placing two implants for a more extensive montage. This commercially available system in this bilateral cortical impact swine model may be useful to a wide range of investigators studying epileptogenesis in PTE.

## Significance Statement

Post-traumatic epilepsy (PTE) is a cause of significant morbidity after traumatic brain injury (TBI) and is often drug resistant. Robust, informative animal models would greatly facilitate PTE research. Ideally, this biofidelic model of PTE would use a species that approximates human brain anatomy, brain size, glial populations, and inflammatory pathways. An ideal model would also incorporate feasible methods for long-term video EEG recording required to quantify seizure activity. Here, we describe the first model of PTE in swine and describe a method for robust long-term video EEG monitoring for up to 13 months post-TBI. The relatively easy “out-of-the-box” radiotelemetry system and surgical techniques described here will be adaptable by a wide array of investigators studying the pathogenesis and treatment of PTE.

## Introduction

Post-traumatic epilepsy (PTE) is the most common form of acquired epilepsy, which is often refractory to treatment. PTE is usually defined as two seizures occurring at least 2 weeks after the traumatic brain injury (TBI) event. In humans, the latent period before the development of epilepsy can last several months or longer (Annegers et al., 1998). The mechanisms of epileptogenesis during this latent period are unknown. Models of PTE have been mainly restricted to variations of rodent impact models where an impact is made to the thin cortex overlying the hippocampus (impact, fluid percussion injury, weight drop; Pitkänen and McIntosh, 2006; Ostergard et al., 2016; Keith and Huang, 2019) with very limited work in gyrencephalic species (Friedenberg et al., 2012; Steinmetz et al., 2013).

The majority of human PTE is neocortical in origin (Gupta et al., 2014). Although processes that are widely hypothesized to be epileptogenic occur in rodent neocortex after trauma (Jin et al., 2011), to date it has not been possible to develop a robust neocortical PTE model in rodents (Bolkvadze and Pitkänen, 2012; Pitkänen et al., 2015). This may be because of the small size of the rodent brain, which results in significant epileptogenic hippocampal injury when the neocortex is damaged by trauma (Komoltsev et al., 2020), or it may reflect a relatively high threshold for the development of stable, chronic epilepsy in the rodent neocortex (Chang et al., 2004; Cela et al., 2019).

The interspecies differences in brain anatomy may also underlie the significant species differences in responses to therapy. In TBI, over 150 therapies have been shown to reduce lesion volume in rodent models but have failed to demonstrate efficacy in humans or in swine (Margulies and Hicks, 2009). Differences in brain anatomy including location of the hippocampus (direct mechanical trauma in rodent impact models vs secondary cascades or diffuse TBI in humans), variability in the abundance of white matter and white matter injury, variation in the pathoanatomic character of the lesion developing from the injury (cavity in rodents vs a remodeled area with thick gliotic scarring in gyrencephalic species), the population and characteristics of the glia (Azevedo et al., 2009; Herculano-Houzel, 2009; Khakh and Deneen, 2019; Khrameeva et al., 2020), differences in the matrisome (Pokhilko et al., 2021), the degree of genetic variation among individual subjects of a given species, and immune response differences (Seok et al., 2013; Warren et al., 2015) all are host factors that may affect the development of PTE among species, and thus may affect our understanding of the development of PTE in humans. Indeed, brain size and the duration of the latent period are positively correlated (Lillis et al., 2015). To design therapeutic interventions that may prevent PTE in humans, the constellation of injuries that occur in humans must be modeled in a brain more similar to humans. Models using gyrencephalic species may bridge this gap.

Simplified *ex vivo* models of PTE such as slice preparations are also available (Berdichevsky et al., 2012; Goldberg and Coulter, 2013; Lillis et al., 2015). Because of the severity of injury (complete transection of the hippocampus at 350 μm intervals), 100% of explants develop medically intractable PTE (Lillis et al., 2015). These preparations are very amenable to longitudinal microscopy studies (Lillis et al., 2015; Lau et al., 2022), but they are based on rodent hippocampi, not neocortices. Further, the complete penetrance of epilepsy complicates studies of epileptogenic mechanisms.

A technical barrier in the adoption of large-animal PTE models is the need for reliable, long-term electroencephalogram (EEG) monitoring because of the longer latent period compared with rodents (Lillis et al., 2015). Long-term monitoring of large-animal models of epileptogenesis has been limited to date. Nonhuman primates have been used for long-term EEG monitoring but are expensive (Vuong et al., 2020), and the use of livestock species for research is more acceptable to the general public perception than nonhuman primates or companion animals. Here, we describe extreme long-term monitoring of a swine model of PTE using a video EEG radiotelemetry system. We discuss the advantages and disadvantages of a contusion model in swine monitored with a commercially available video EEG radiotelemetry system enabling real-time analysis.

## Materials and Methods

In conducting research using animals, the investigators adhered to the laws of the United States and the regulations of the Department of Agriculture.

### The radiotelemetry system

The Data Sciences International (DSI) PhysioTel Digital radiotelemetry system enabled transmission of EEG data from subcutaneous implanted electrodes in real time via Bluetooth to a transceiver connected to a communication link controller (CLC) that managed the EEG implants and relayed digitized data to a computer. Video was recorded and synchronized to the EEG data. At the peak of the study, up to six swine were recorded at the same time.

**Figure 1.**
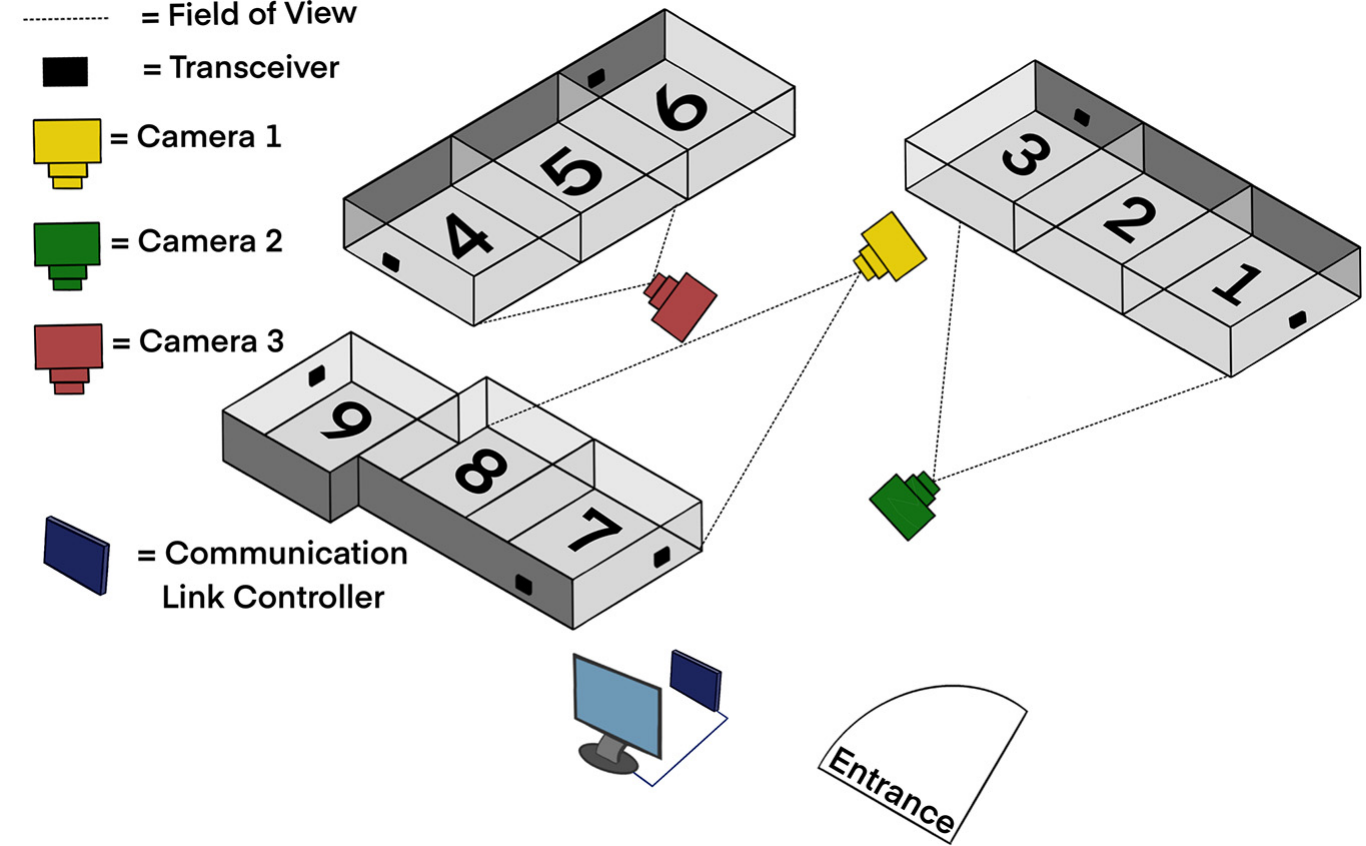
Diagram of the video EEG digital radiotelemetry monitoring unit. The transceivers receive EEG data from the implanted transmitters via Bluetooth, which is connected via cables (not shown in diagram) to the communication link controller, which then communicates via cable to the computer that stores the data outside of the telemetry room. With three cameras, up to six pigs could be recorded with video at the same time with large pigs taking two pens in a total of eight pens. Pigs were separated for their weekly night of video EEG recording.

All EEG and video acquisition hardware was installed via the manufacturer instructions in the *DSI Implantable Telemetry System Manual* (Data Sciences International, 2017; Fig. 1). The CLC allows communication between the implants and computer by discovering implants, assigning frequencies, and relaying digitized EEG data via an ethernet connection to the computer. The CLC can manage up to six EEG transmitters at a time. The CLC was housed in the animal room in a stainless steel lock box to prevent water damage during cage cleaning. The CLC was connected via ethernet cables tunneled through the ceiling to the data acquisition computer located just outside the animal room. The EEG transmitter communicated to the CLC via transceivers. Per bank of pens, two to three transceivers were secured to the animal cage walls at a height that was inaccessible to the animals while also not blocking the field of view of the video cameras. The transceiver cables were routed along the outer perimeter of the cages and along the room walls to the CLC. It is recommended to place transceivers at right angles to one another to minimize areas of poor signal reception and prevent signal drop-off (Data Sciences International, 2017). Three video cameras (AXIS M1145-L Network Cameras, Axis Communications) were installed on the ceiling 2–3 feet away from the bank of pens and placed in a way that maximized field of view at each of the three banks (Fig. 1). Each camera recorded two independently housed or three socially housed animals at a time. Each camera was enclosed within an acrylic box [a modified basketball display case: 10.25 inches; height and length; The Container Store (not provided by Data Sciences International)] to protect the cameras from water damage during pen washing. It was opened at night to allow video recording in infrared mode. All cables from the video cameras were routed along the ceiling to an ethernet data port in the animal room to an ethernet data port outside the animal room and connected to the data acquisition computer. Feeders fixed to the front of the cage were removed and replaced with rubber dishes on the pen floor to increase visibility with video recording. Video was recorded using MediaRecorder 4.0 software (Noldus). A key synchronization step was required to synchronize the video and EEG acquisition software using the “Network Time Protocol” following the manufacturer instructions so that the time stamp on the video matched the time of EEG recording, allowing the analysis of EEG and video in synchrony in NeuroScore. The synchronization was measured via the manufacturer instructions: “A TTL [transistor–transistor logic] pulse was sent simultaneously via split cable into the acquisition interface and adapted telemetry biopotential channel was acquired via the telemetry receiver.”

**Figure 2.**
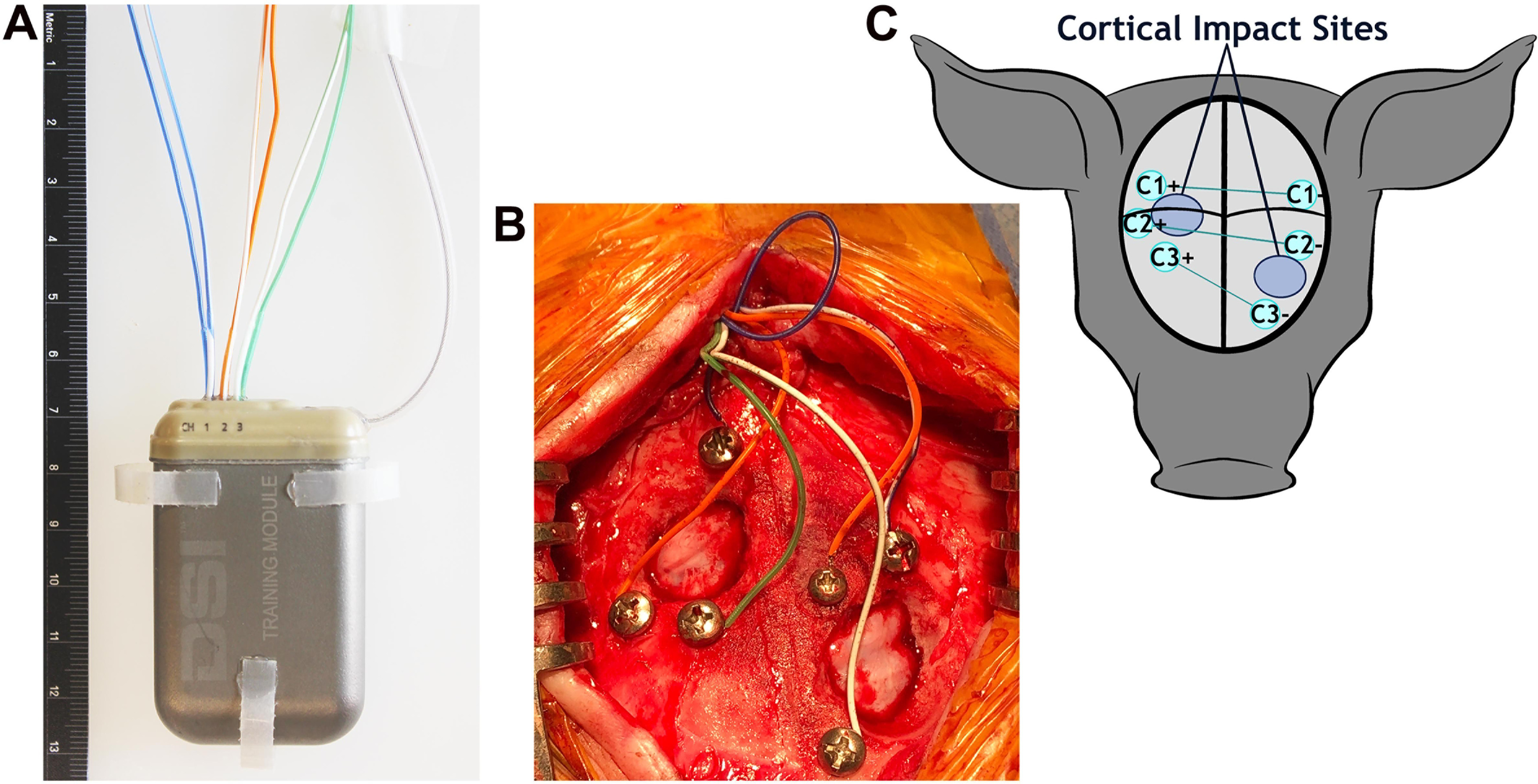
EEG implant and montage. ***A***, The 59 × 38 × 15 mm EEG transmitter with electrodes exiting the top of the implant, the antennae projecting off the right of the implant, and tabs on the sides and bottom that are used to suture the implant in place. ***B***, Pigs received bilateral cortical impact through the burr holes. The electrode array before the application of dental cement. ***C***, A schematic of the three-channel bipolar montage with electrode sites (teal circles) centered around the sites of cortical impact (large blue circles). Black lines represent the sagittal and coronal sutures.

The PhysioTel Digital L03 series implant (three channels; Fig. 2*A*) was used in this study in conjunction with the Ponemah Data Acquisition software (Data Sciences International). The acquisition frequency was 500 Hz. The L03 series implant had six biopotential leads with no common leads. Instead, each pair of the biopotential leads was coupled into an instrumentation amplifier resulting in differential inputs to three channels. Each biopotential channel had a common mode rejection ratio of −40 dB or better at test frequencies of 0.5 and 10 Hz. The common mode signal applied to the biopotential channel was generated with respect to the implant housing connection (ground). There were no hardware filters. A software filter that was a 30th-order finite impulse response was applied. A low-pass filter for 150 Hz was used to guarantee accurate signal acquisition and channel bandwidth of 0.5–100 Hz; this was verified by testing with a signal or function generator. No low-frequency signal was filtered. A Blackman window was used for calculating coefficients for the filter designed as a windowed-sinc filter (Troy Velie,Data Sciences International, personal communication).

In addition to the biopotential lead signals, the implant also provided temperature measurements as well as activity measurements measured via a three-axis accelerometer. From the manufacturer instructions: “The three-axis accelerometer provides acceleration data along the *x*-, *y*-, and *z*-axes, relative to the orientation of the implant. Acceleration for the *x*-, *y*-, and *z*-axes was reported as a value from an analog-to-digital converter. A range of at least −7 Gs to +7 Gs was provided, with a corresponding output from ∼0 to 4095. A value near 2047 was displayed when zero acceleration for a given axis was sensed when in a steady, neutral alignment (orthogonal) to earth’s gravitational field. The displayed sampling rate for the *x*-, *y*-, and *z*-axis acceleration data was 10 Hz. Along with the values from each axis of the accelerometer, Ponemah also reports an activity value calculated from the accelerometer axes…” (Data Sciences International, 2017). The accelerometer data were used to detect movement to screen for convulsions compressing the length of video required for manual screening.

The manufacturer estimate for battery life for the implant was 90 d. To enable prolonged monitoring, to conserve battery life, the implant was turned on and off using a strong magnet swiped over the implant and could also be turned off via the acquisition software.

Animals were housed in a temperature-controlled animal facility with 12 h light/dark cycles. Video EEG was recorded following a schedule that maximized vivarium recording capacity. Animals were warehoused at an off-site facility for additional space. When on-site, the animals were recorded with EEG and video or video only at least every other week and as space permitted. Animal facility staff were given a recording schedule and moved animals per the schedule.

### Surgery for EEG implantation and cortical impact

Male, castrated Yucatan minipigs (*N* = 17; Sinclair Bio Resources) were implanted at the mean ± SD age of 4.92 ± 0.37 months at a weight of 21.5 ± 2.8 kg (Table 1). Grain pellets were removed the evening before surgery, and the swine were fasted for 12 h. Water was available at all times. The animals received a Hibiclens bath the day before surgery and the morning of surgery before anesthetic induction where the animals were gently sprayed with warm water and scrubbed with ∼5 ml of Hibiclens into a lather. Diazepam was administered (2–4 mg/kg, p.o.) in simple syrup 30–45 min before anesthetic induction. Swine were sedated with a preanesthetic mix consisting of ketamine (20 mg/kg, i.m.), xylazine (2 mg/kg, i.m.), and atropine (0.03 mg/kg, i.m.). Pigs >50 kg were sedated with TELAZOL (2.2–4.4 mg/kg, i.m.), xylazine (2 mg/kg, i.m.), and atropine (0.03 mg/kg, i.m.) to minimize total injection volume.

**Table 1:** Ages, weights, and number of pigs

Event	Age (months)	Weight (kg)	Time after cortical impact (months)	Number
Cortical impact or sham surgery	4.92 ± 0.37 (3.1–5.5)	21.5 ± 2.8 (16–25)	N/A	*N* = 13: 10 injured, 3 shams (8 implanted at the time of cortical impact)
Implantation of EEG for those not implanted at the time of cortical impact	10.45 ± 1.2 (8.4–11.6)	43.2 ± 2.6 (36–47.5)	6.43 ± 0.64 (4–6)	*N* = 5
Killing after development of PTE was detected	13.1 ± 4.1 (7–16)	68.0 ± 12.7 (53– 84)	9.2 ± 2.5 (5.6–11.4)	*N* = 4
Scheduled killing: did not or did not develop PTE	17.6 ± 0.7 (16.3–18.5)	82.3 ± 9.4 (62– 92)	12.4 ± 1.0 (11.5–14.2)	*N* = 9

Values are average ± SD (range), unless otherwise indicated.

Pigs were transported to the operating room under 3–5% isoflurane, and oxygen was delivered via nose-cone mask while monitoring oxygen saturation and heart rate using a handheld pulse oximeter unit. Before entering the operating room, swine were shaved at incision sites, feet were wrapped with Vetrap (3M Health Care), and eye lubricant was placed along the inner edge of the eyelid then closed with a piece of tape followed by a full Tegaderm patch (3M Health Care). Xeroform Petrolatum Gauze (Covidien) was inserted into the ears. The head, ears, and neck were washed with 2% chlorhexidine cloths, then the swine were moved onto the operating table.

An intravenous catheter was placed in an ear vein. Vancomycin (10–20 mg/kg) was infused intravenously over 30–60 min followed by saline (2–4 ml/kg/h, i.v.). Swine were intubated and mechanically ventilated with isoflurane titrated to 1–2% mixed with medical air. Ventilation was adjusted so that end-tidal CO_2_ was maintained between 35 and 45 mmHg with a peak pressure of 20–25 mmHg. Core body temperature was measured via a rectal probe and maintained at 37–39°C using a heating pad and Bair Hugger forced air blanket. Swine received an infusion of saline (2–4 ml/kg/h). Blood pressure, as measured via a cuff on a hindlimb, was maintained at >45 mmHg. Saline boluses (2–4 ml/kg, i.v.) were administered for hypotension [mean arterial pressure (MAP), <45 mmHg]. Epinephrine (5 μg/kg, i.v.) was administered if saline did not successfully increase MAP. End-tidal CO_2_, oxygen saturation, blood pressure, heart rate, and core body temperature were monitored and recorded every 15 min. Preinjury and 2 h postinjury blood was collected intravenously or via superior vena cava venipuncture for later analysis. Blood was collected 24 h postinjury via the superior vena cava. Buprenorphine (0.02 mg/kg) was administered intramuscularly 15 min before the first incision.

The ears were wrapped with sterile Vetrap. Tegaderm was placed over the top of the snout, over the eyes, and around the ears, creating a continuous perimeter of Tegaderm around the surgical site. The swine was positioned in sternal recumbency such that the head and neck were accessible, and rolls of absorbent pads were placed underneath the swine to reduce pressure on the abdomen. The surgery was performed in a single scrub position. The scrub was performed with a separate scrub pack after the surgeon scrubbed and gowned. The incision sites (head and right side of neck) were prepped using surgical sterile technique using 70% ethanol followed by betadine using gauze held with a dedicated scrub hemostat, then with ChloraPrep, which was allowed to dry (Becton Dickinson).

After prepping, sterile Steri-Drapes (3M Health Care) were placed around the incision sites followed by Ioban (3M Health Care). Last, a large Tiburon Split-Sheet Sterile Drape (Cardinal Health) was placed over the entire surgical area. The drape was clipped to an intravenous stand in front of the head of the animal such that the endotracheal tube remained in view for adjustment when necessary and to test mucous membrane and jaw laxity for anesthetic plane.

To minimize the risk of infection with implants, all instruments were autoclaved, and gas-sterilized instruments were not used. Bupivacaine (1.5–2.5 mg/kg) was administered subcutaneously at the head incision site. The first skin incision was made along the sagittal midline from above the snout to the crown of the head. The skin was detached from the periosteum to expose the skull. The sagittal and coronal sutures were identified, and a Hudson drill was used to make a burr hole on the right coronal suture over the rostral gyrus. A dural separator was used to detach the dura from the underside of the skull. Bone rongeurs were then used to expand the burr hole to ∼2 cm in diameter. Hemostasis was obtained with sterile bone wax.

The cortical impactor guide was secured to the skull at each burr hole (Duhaime et al., 2000). The cortical impact device was screwed into the guide until the 1.07 cm in diameter tip was just touching the surface of the dura. The indenter was then deployed (over 4 ms) over the closed dura with an indentation velocity of 1.7 m/s (Duhaime et al., 2000). In a similar manner, a second burr hole was made on the left, rostral to the coronal suture to expose the very rostral portion of the brain. The prefrontal cortex is the somatosensory cortex that represents areas of the face and portions of the mouth; the rostral gyrus is the somatosensory cortex of the snout (Craner and Ray, 1991; Missios et al., 2009). Cortical impact was performed at two sites to potentially induce a greater rate of PTE than one site would. The offset of contusion locations on the two sides was chosen to minimize any functional disability that might be caused by bilaterally symmetric lesions.

A Stille Bone Hand Drill (Sklar) with 1.5 and 2.0 mm drill bits was used to drill six holes for skull screws, three on each side around the area of the burr hole (Fig. 2*B*,C). Everbilt Pan Head Philips Stainless Steel #4 screws [Home Depot Product Authority (not provided by DSI)] with a 2.85 mm head diameter were filed down to varying lengths between 5 and 15 mm to accommodate variable skull thickness. Screws were threaded into the drilled holes with a surgical screwdriver. A dural separator was used during installation of the screws to verify that each screw was placed through the skull and in contact with the dura.

Bupivacaine (1.5–2.5 mg/kg) was administered subcutaneously at the neck incision site on the right side of the neck ∼6 cm posterior to the ears and 6 cm lateral to the midline. An ∼5 cm neck incision was made with a different set of sterile instruments that had been set aside and not previously used. A pocket was open under the skin via blunt dissection beneath the subcutaneous fat or under the trapezius muscle until it was slightly larger than the implant. The sterile EEG transmitter was removed from the sterile packaging using a new set of sterile gloves. The edges of the skin were draped with a second set of Steri-Drapes and gauze packed around the edge of the incision such that the implant did not touch the skin. The EEG transmitter was implanted either under the subcutaneous fat or under the trapezius muscle and sutured into place with the implant tabs. The EEG implant biopotential leads were tunneled under the skin from the implant site to the caudal end of the head incision using a Nelson 35 French trocar (Sklar). The neck incision was irrigated copiously with sterile saline, and the incision was closed with interrupted 2–0 PDS Suture (Ethicon) in the subcutaneous layer, and 3–0 monocryl (Ethicon) subcuticular suture followed by LiquiVet Rapid tissue adhesive (Oasis Medical).

The biopotential leads were trimmed to a size long enough to reach the skull screws while leaving enough additional length to allow for pig growth and movement. Leads (skull screws with bipotential leads wrapped around) were placed in a bipolar montage to the right and left focused around the cortical impact sites (Fig. 2*B*,*C*). While the burr holes for cortical impact were placed via skull landmarks (described above), the skull screws/electrodes were placed adjacent to the burr holes as skull thickness allowed with electrodes for channel 1 being most caudal, channel 2 being in the middle, and channel 1 being most rostral. In the Yucatan pig, the skull thickness rapidly increases on the sides with a relatively flat top. There was limited ability to place screws on the side of the skull because of the thickness of the skull, resulting in some differences in placement around the burr holes among subjects but keeping the orientation of channels the same among subjects. Approximately 5 mm of the silicon insulation was stripped from the biopotential leads, and the exposed lead was wrapped around the shaft of each screw and secured using silk suture. The screw was then tightened to secure the lead to the skull with the screw in contact with the intact dura below. Screws and leads were required to be low profile to allow skin closure. Maxcem Elite dental acrylic (Kerr) was applied to completely cover the exposed screws and leads to ensure electrical isolation from surrounding tissues (Data Sciences International, 2012). Once the dental cement set, the cortex was irrigated with sterile saline and the incision was closed with interrupted 2-0 PDS suture for the subcutaneous layer and 3-0 monocryl running subcuticular skin closure followed by skin adhesive.

To optimize the time interval over which epileptogenesis could be observed, a subset of pigs that received cortical impact did not receive an EEG implant until 6.43 ± 0.64 months post-cortical impact (*n* = 5; Table 1), though they were video recorded before the implantation of the EEG transmitter. The intended time to implant was 4 months post-cortical impact but was delayed because of lockout from our facilities because of the COVID pandemic lockdown in 2020.

Sham pigs (*N* = 3) underwent the same surgical procedure, including installation of the EEG transmitter and skull screws, except the cortical impact device was not deployed. The scrub and surgery required 6 h with an additional hour required for recovery.

### Postsurgical recovery and monitoring

Isoflurane was reduced, and the pig encouraged to breath by allowing end-tidal CO_2_ to increase. Buprenorphine was administered (0.025 mg/kg, i.m.) and fentanyl transdermal patches (1–4 μg/kg/h) were placed on the lower back for pain management for 72 h after surgery. The animal was then transferred to the animal facility under 1–2% isoflurane and extubated. The animal was monitored until ambulatory. Antibiotic ointment (2% Mupirocin ointment, Taro) was applied to both skin incisions the first day after surgery to prevent infections. Prophylactic cephalexin (10–20 mg/kg) was administered orally three times a day for 7 d postoperatively. The animals received twice-daily evaluations for 3 d postoperatively and five times a week until the incisions were fully healed. Subjects were not transferred to a satellite facility until full healing was achieved ∼1 month postsurgery.

Though the areas receiving cortical impact were expected to be clinically silent, swine were often somnolent the day after surgery, sometimes had temporary difficulty with coordination/movement of the front left leg that resolved in the day or two after surgery. Though no formal consistent vision testing was performed, temporary limitations in vision were suspected as some swine receiving bilateral cortical impact had absent menace responses, startled to touch, and tripped over their food bowl. This behavior was not observed in sham pigs. The signs of vision impairment resolved by postsurgical day 1 or 2.

The site of implantation in the neck displayed significant tissue swelling in the first 3–5 d postsurgery but resolved thereafter. Less swelling was observed when the implant was placed under the trapezius muscle versus under the subcutaneous fat.

### Animal husbandry procedures

In these long-term experiments, the animal enrichment team provided swine with regular stimulation and socialization. Staff provided a new toy or activity daily. Once swine reached 50 kg, they were placed in two pens to enable space to accommodate their larger size. Swine were introduced to one another over time so that compatible swine were socially housed during days of video-only recordings. The specific individuals in each 24 h video were logged in a spreadsheet where they were identified by physical markings or the presence of two ear tags versus one. When pigs were scheduled to have EEG and video recorded, they were placed in an individual pen. Isolating the pig during EEG recording was crucial in later analysis as it was difficult to assess whether an event was real electrographic activity or artifact because of the movements of their pen partner. Metal feeders in front of the cage were removed and replaced with rubber feeders during healing from surgery and during recording as they obstructed the view from the video cameras. Placards were posted in the room instructing staff where to scratch the pigs to avoid interfering with the incision sites. Swine received regular food treats including yogurt and apricots, and often did not require restraint for preanesthetics because of acclimation with our study staff.

Animals that received a second ear tag for identification purposes for recording with social housing or required hoof trimming (approximately every 3 months) were anesthetized for these procedures. Swine were removed from feed 12 h before anesthesia and were anesthetized using the preanesthetic mix described above (or, once at a weight >50 kg, swine were given TELAZOL, 2.2–4.4 mg/kg, i.m.) and then anesthetized under 3–5% isoflurane and oxygen. Hair was clipped if necessary and was cleaned with alcohol or betadine. The tags were cleaned with alcohol or betadine and inserted into the outer portion of the ear while avoiding the outer cartilage supporting the ear and the central ear vein and other large veins using an ear tag applicator and/or the hoofs were trimmed. After the procedure was completed, isoflurane was reduced, and the pig was encouraged to breathe and recovered.

Yucatan skin required regular management. To treat dry, itchy skin, staff applied mineral oil to the body of the pigs daily until their skin was healthy, and thereafter, weekly to maintain healthy skin. Regular oiling prevented the animals from scratching their implant site with their hindlegs or against the side of the cage. Several Yucatan pigs spontaneously developed blisters and open lesions over the dorsum consistent with bullous pemphigoid as previously described in this strain (Mirsky et al., 2000; Olivry et al., 2000). The open lesions were cleaned with diluted chlorhexidine gluconate and treated with topical antibiotics as needed. Many displayed allergic reactions to bacitracin, neomycin, and polymyxin topical antibiotics as well as to unidentified substances during surgeries. Allergic reactions were limited to the skin, were self-limited, and did not require treatment.

A limitation of this study concerned the logistic issues of housing these large animals at our institution for up to 14 months. A great deal of planning and communication with the animal facility was needed to successfully accommodate these animals. However, given that the animal facility had limited space, many of our study animals had to be sent out to an outside animal facility, which resulted in week-long to month-long gaps in video EEG recording for some animals.

### Euthanasia and brain collection

After developing PTE or at 12–14 months post-cortical impact (Table 1), pigs were withdrawn from feed for 12 h and given diazepam (2–4 mg/kg, p.o.); TELAZOL was administered (2.2–4.4 mg/kg, i.m.) 30 min later, then the pigs were deeply anesthetized with 3–5% isoflurane and intubated with a 9–10 endotracheal tube. The pig was moved by four staff members on a pig board, transferred to a hydraulic lift, then to a motorized cart, and moved to a downdraft necropsy table. After ensuring a surgical plane of anesthesia, swine were killed via exsanguination by transcardiac perfusion with 0.9% saline and 10% formalin. The skull was opened with a bone saw, and the brain, including the olfactory bulbs and 2 cm of spinal cord, was collected. The time required for killing, carcass disposal, cleanup, and brain removal was 8 h. The brain was weighed and postfixed at 4°C for 5–7 d. The cerebral hemispheres were coronally sliced. Blocks were paraffin embedded and stored in sealed containers at room temperature for future investigation.

## Results

### Electroencephalographic recordings

To date, this is the first published model of any type of epilepsy in swine and provides the longest-term recording of which we are aware. The system was relatively easy to set up.

Video EEG was recorded until PTE developed or for 12 months (maximum of 13 months) in 13 subjects (10 injured pigs, 3 sham pigs). The average duration from the time of EEG implantation to the end of the experiment among all animals was 11.5 months, achieving very long-term monitoring.

**Figure 3.**
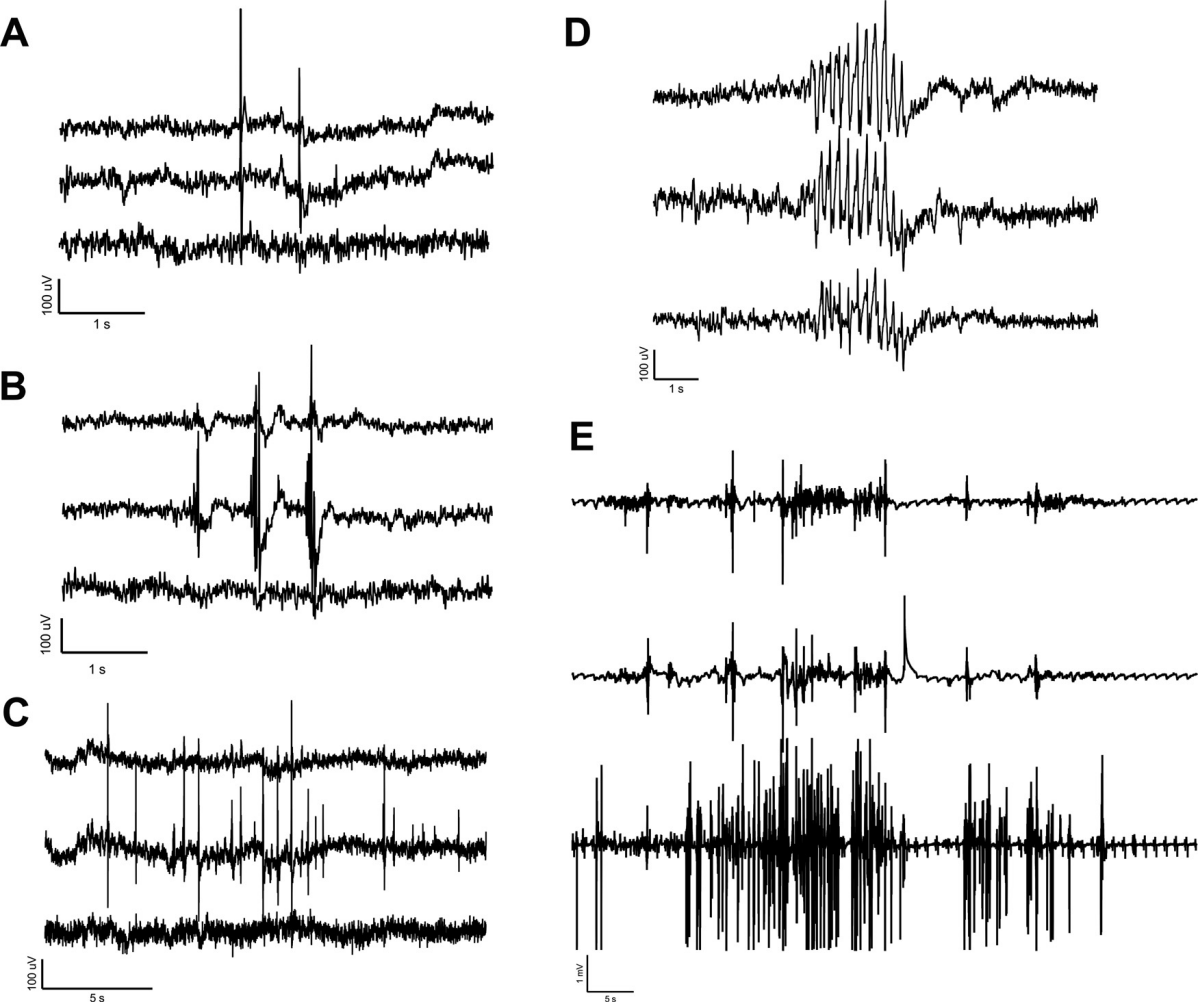
Interictal spikes and electrographic seizure on our three-channel array (channel 1, top trace; channel 2, middle trace; channel 3, bottom trace; Fig. 2*C*, montage). ***A***, A simple spike. ***B***, Complex spikes. ***C***, A train of complex spikes. ***D***, Clusters of waves with spikes. ***E***, An electrographic seizure accompanied by tonic-clonic convulsions.

A portion of the pigs receiving cortical impact developed epileptiform spikes, electrographic seizures, and convulsions. The rate of epilepsy and analysis of interictal epileptiform discharges in relation to the onset of convulsions will be published in a separate manuscript. The EEG of injured pigs that developed PTE showed a wide array of epileptiform discharges similar to those seen in human patients. Simple and complex spikes, sharp waves, spike trains, clusters of waves and spikes, and electrographic seizures were recorded in multiple injured animals before and after developing convulsions (Fig. 3).

**Figure 4.**
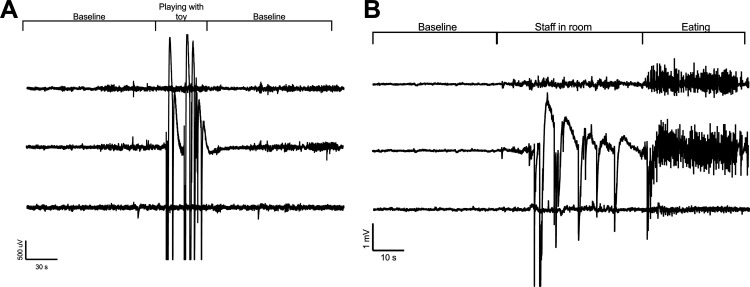
Artifact on our three-channel array (channel 1, top trace; channel 2, middle trace; channel 3, bottom trace; Fig. 2*C*, montage). ***A***, High-amplitude artifact was observed while the animal was playing with a toy or during a vigorous head shake (not shown). ***B***, High-amplitude artifact in response to anticipation of feeding (including jumping up on side of pen) followed by low-amplitude muscle artifact of eating.

Similar to ambulatory EEG systems in rodents and humans, movement artifacts occurred during large movements verified by the synchronized video (Fig. 4), but the system was robust and did not display movement artifacts from minimal muscle movement. Large-amplitude and high-frequency sharp spikes saturated the EEG signal when staff fed the swine and they would jump up on the side of the pen before receiving their food. Large-amplitude artifacts occurred when animals jumped up on the sides of the pen to greet neighboring animals or facility staff, playing with enrichment toys (usually involving rapid, repetitive head movement), and during headshakes. Low-amplitude muscle artifacts occurred with eating. However, most low-speed activities such as drinking water, moving the head around during normal voluntary movement, walking around the pen, and gentle sleep rocking were not detected on the EEG. As a result of these factors, periods where the animals were lying down or doing minimal physical activity were optimal for EEG analysis.

### Behaviors

**Figure 5.**
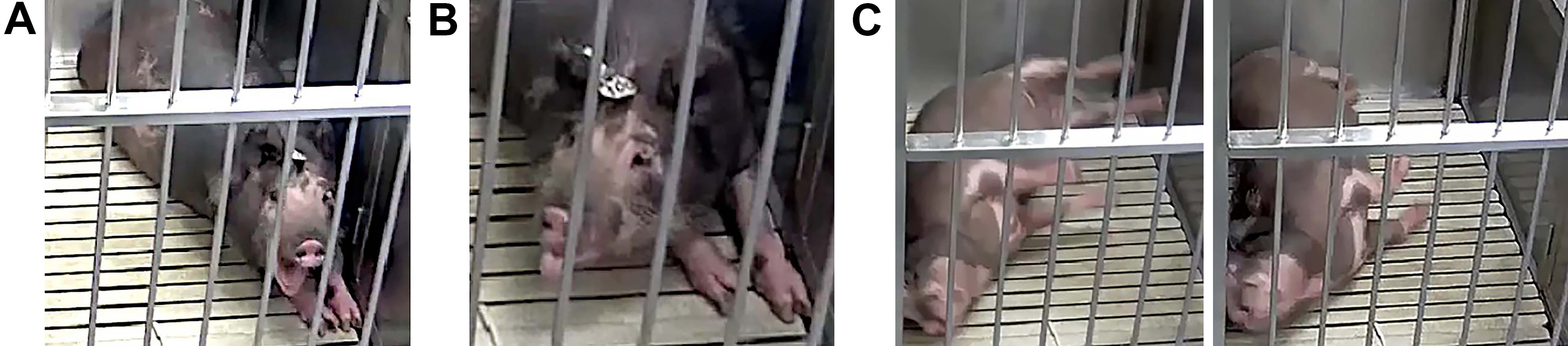
Still images of behavior recorded on video. ***A–C***, This subject displayed an array of stereotypical automatisms including yawning (***A***, ***B***) the tongue out before tonic-clonic convulsions (***C***). ***C***, One round of a tonic-clonic convulsion with legs extended (left) and then legs relaxed with the head back (right) with the animal laying on its side.

Throughout the study, injured pigs were observed to have a variety of stereotypical peri-ictal behavioral repertoires. These behaviors included staring spells, head nodding and shaking, instances of forelimb clonus and tonic extensions, lip smacking and licking, and tonic-clonic convulsions followed by postictal stillness (Fig. 5). Some of the epileptic animals seemed aware of the oncoming epileptic episodes such that they would gently lower themselves to the ground or lean against the side of the cage to lower before convulsing. Most pigs would convulse during emergence from sleep. In only one instance was a pig observed to drop suddenly. The analysis of semiology for each pig with PTE as well as non-peri-ictal behavior specific to pigs with PTE versus normal swine behavior will be published separately. Unlike rodents with a Racine scale of level 5, no individual was observed to be apneic, and none reared up on its back legs or died from convulsions. In collaboration with our large-animal veterinarians, no convulsive event was deemed to endanger animal welfare. The only injuries observed were abrasions on the sides and legs from rubbing on the floor during convulsions.

### EEG implant and recording limitations

A limitation of this telemetry unit is the limited battery life of 90 d, which limits recording to one to two times/week in subjects in which several months may be required for the development of PTE. Three implants indicated several days of battery life left but failed to record at the very end of battery life; actual battery life was typically 80 d when switched on and off regularly. One of 13 EEG transmitters completely stopped functioning 20 weeks into the study. Despite having ∼78 d of battery life remaining, the implant failed to turn on. The subject was changed to a video-only recording schedule. An attempt was made to use the manufacturer crimping tool to switch out an implant where the battery was expended and splice the existing biopotential leads to a fresh new device. However, this surgical procedure was not possible as wires were encapsulated by the body and were difficult to remove without damaging. The animal was switched over to a video-only recording schedule.

Staff compliance with the recording schedule was high, while compliance with removing metal feeders during video EEG recording was low. Additionally, compliance with keeping the transponder so it was not located on the pen between the pig and the video camera was also low. An auditing schedule and additional placards in the recording room could improve compliance and improve the quality of the video acquired.

### Montage limitations

Though we were able to quantify epileptiform activity, it is not possible to remontage the array, limiting our ability to identify a specific seizure focus with three biopotential channels. The acquisition bandwidth was limited to 0.5–100 Hz, thereby limiting the ability to acquire infraslow frequencies or high-frequency oscillations (<0.5 or >100 Hz, respectively).

**Figure 6.**
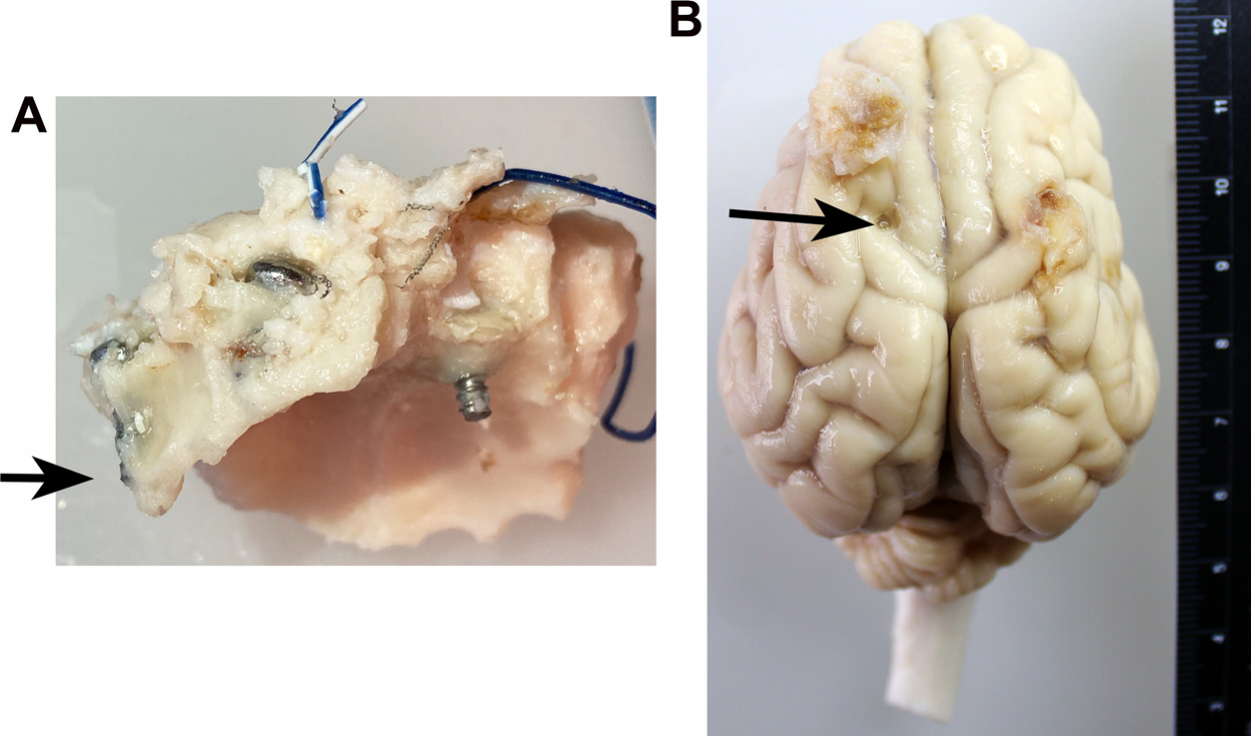
The evolution of screw location. ***A***, ***B***, Because of the significant accretion of skull over time with the development of the nuchal ridge, screws initially placed to just contact the dura eventually were completely embedded within the skull (***A***; arrow), exposed to air in the sinuses, or embedded within the brain (***B***; arrow; ruler units = centimeters). Screw indentations were observed in both swine that did and did not develop PTE and did not appear to be a cause of PTE.

There was significant accretion of skull bone over time that resulted in unpredictable shifts in screw placement and dura contact from implantation to the end of the study. At age 17 months, the nuchal ridge is very large over the crown of the head and paranasal sinuses cover the skull on the sides the skull covering the rostral portions of the brain. Screws placed most caudally were most affected by skull thickening often becoming completely embedded in skull bone (Fig. 6*A*), while rostral screws sometimes were found to be exposed to air in the sinuses and were no longer in contact with the dura. Some screws were found to leave shallow indentations in the brain, transversing the dura in some cases (Fig. 6*B*), but were not associated with the incidence of PTE: pigs both with and without PTE exhibited screw indentations. Despite the alterations in screw location, we were able to record long term from the pigs in all but one subject. In one subject, the ability to detect spikes was absent at ∼34 weeks postinjury because of the screws becoming completely embedded into the skull.

### Morbidity and mortality

Early in the project, we encountered problems with infections that were solved by adapting surgical sterile technique protocols from the human operating room along with other strategies (Table 2). The EEG transmitter is large and provides an extensive area ripe for the development of biofilm. Implementation of the protocols in Table 2 and regular pig oiling completely ended the infection issue. As many measures were implemented at once, it is impossible to identify which were the key factors, but once the infection problem had abated, one pig that failed to receive its presurgical baths developed a methicillin-resistant *Staphylococcus aureus* (MRSA) infection. Therefore, presurgical baths may be key to preventing infections. Specifically, four pigs were infected at the implant site, requiring early killing, and were excluded from the study. The infection in two pigs was because of MRSA infections that developed rapidly (2 weeks) after implantation. Two pigs had infections that presented later at the implant site at 7 and at 14 weeks after implantation though postsurgical swelling was greater than normal. These infections were positive for beta-hemolytic *Streptococcus*, *Staphylococcus schleiferi*, and *Streptococcus porcinis*. In one instance, the delayed infection may have resulted from a superficial scratch early after implantation that eventually abscessed. The scratches may have resulted from the pig scratching dry, irritated skin. Thereafter, swine were on a regular oiling schedule to prevent itch and thus scratching.

**Table 2:** Procedures and medications initiated to prevent infection above and beyond standard large-animal survival surgery standards

Measures	Description
Preoperative measures	All surfaces of the operating room including the walls and ceiling were sanitized with Quatricide PV-15 (Pharmacal) prior to each surgery
	Swine received a Hibiclens (chlorhexidine gluconate) bath over the entire body the day before and the day of surgery
	All hair clipping was performed before entering the OR
	Feet were wrapped with Vetrap before entering the OR
	Xeroform Petrolatum gauze inserted in the ears
	Head, ears, and neck were washed with 2% chlorhexidine wipes before entering the operating room
	Staff replaced all personal protective equipment before entering OR
Additional scrub and drape measures	Ears were wrapped with sterile Vetrap
	Tegaderm was placed over the top of the snout, over the eyes, and around the ears creating a perimeter around the surgical site on the head
	A dedicated scrub pack was used separate from the instrument pack with the surgeon rescrubbing/regowning after scrub
	Three-step scrub instead of two-step scrub: incision sites prepped with 70% ethanol then with betadine using gauze held with a dedicated scrub hemostat, then with a ChloraPrep wand
	Three layers of drapes instead of two: sterile Steri-Drapes were placed around the incision sites then site was covered by Ioban then with a large split-sheet sterile drape placed over the entire surgical area
Prophylactic antibiotics	Vancomycin (10–20 mg/kg, i.v.) infused over 30 min prior to the first incision
	Cephalexin (10–20 mg/kg, orally) three times/d for 7 d
Other	Using dedicated instruments for the neck site (not used at the head site) and a new pair of surgical gloves to handle the EEG transmitter
	All instruments were heat/pressure sterilized as possible. Chemical sterilization with ethylene oxide was not used
	The EEG transmitter was prevented from touching the skin of the pig by adding new drapes and packing sterile gauze around the incision site
	Surgical sites were heavily irrigated with sterile saline prior to closing
	Postsurgically, the skin of the pig was oiled regularly to prevent irritation and scratching
	Placards placed on the swine pen indicated areas approved for scratching by caretakers avoiding incision sites

OR, Operating room.

## Discussion

Acute EEG recordings in anesthetized swine after various brain insults are relatively simple and are well established (Costine-Bartell, Price et al., 2021), but recording EEGs in awake and restrained and/or tethered swine over the course of several days is more difficult. The standard of EEG in PTE in rodent models of TBI is tethered EEG (Shandra and Robel, 2020) or radiotelemetric EEG (White, Williams et al., 2010). In rodents, the onset of PTE is 21 d (or earlier) to ∼2 months post-TBI. However, in large-brain species, epileptogenesis after TBI develops over several months, requiring a corresponding period of video EEG monitoring and analysis (Lillis et al., 2015). Piglets can be recorded with scalp electrodes for EEG or amplitude-integrated EEG while restrained and kept calm for durations of time similar to those for clinical EEG in humans. Such scalp recordings enable the study of acute effects of TBI or therapies for brain insults (Wang et al., 2014; Atlan and Margulies, 2019; Barata et al., 2019). Similarly, telemetric EEG devices can be temporarily placed on the head, allowing the piglet to ambulate freely while awake or sleeping (de Camp et al., 2018). However, the labor required to apply, record, and remove EEG may be cost prohibitive if extended to several months and may also interfere with normal behavior. The first report of recording from chronically implanted EEG electrodes in swine was up to 3 months (Stromberg et al., 1962). Depth electrodes into the hippocampus have been used up to 6 months in tethered swine with head mounts (Forslid et al., 1986; Ulyanova et al., 2019). Recent advances have been made in telemetry in swine with a DSI implant to record EEG in fully ambulatory EEG in piglets for up to 5 d (Rault et al., 2019). In this instance, EEG was limited to a single channel but was sufficient for power analysis (Rault et al., 2019). Such short-term recordings do not require extensive perioperative, operative, and postoperative methods for success (Rault et al., 2019).

While electrodes are generally resistant to infection (Stromberg et al., 1962), fully unrestricted ambulatory EEG requires an implanted transmitter/battery assembly that comprises a large-volume foreign body that is prone to infection seeded at the time of implantation. The advantage of the fully subcutaneous neck implant is the lack of risk of infection after initial implantation. In contrast, head mounts have a continuous risk of infection because of repeated butting and consequent wound dehiscence. Exteriorized head-mounted systems are preferable in nonhuman primates as they pick at their subcutaneous implants (Vuong et al., 2020) and do not typically head butt. However, swine head butt frequently and thus frequently break exteriorized head-mounted systems, resulting in a constant risk of infection and/or destruction over time. Here we report measures that were successful in preventing infection at implantation and report long-term stability of the fully subcutaneous implant for 12–13 months, allowing for extreme long-term monitoring.

We observed a loss of signal at 34 weeks in one subject and implant malfunction at 20 weeks in another. However, the duration of recording exceeded what has been reported for hippocampal depth electrodes tested in naive swine, where there is a significant loss of oscillation power within the first month followed by persistent loss over 6 months (Ulyanova et al., 2019). Additionally, hippocampal depth electrodes might create more damage than the alterations inflicted by skull screws as they are associated with acute hemorrhage and chronic lesions with activated microglia and gliosis (Ulyanova et al., 2019). Many types of EEG arrays may perturb the system that they record, and, certainly, the integrity of the signal is an issue in all methods of invasive, long-term EEG recording.

Space restrictions resulting in limitations in consistency of video EEG recording for large animals staying for prolonged periods of time could be overcome by installing an additional DSI system at a warehouse facility where staff can manage recording as described here. Swine could be sent to the warehouse recording facility when healed from the surgery (3–4 weeks postsurgery). In this scenario, up to 12 swine could be recorded weekly. Because of collaboration with our animal housing facility administration, a contract with an outside institution was established so that future studies will have the ability to record video EEG at the warehouse site to ensure consistent capture of data.

The advantages of this system are (1) availability of a “kit” where DSI provides most of the equipment, (2) excellent signal-to-noise ratio with algorithms that create reference and ground resulting in minimal movement artifact, (3) the swine freely ambulate and the implant is completely under the skin/not at risk of destruction, and (4) the availability of continuous video with excellent synchronization of video to EEG. The disadvantages of this system are as follows: (1) the skull screws may become embedded in the skull or exposed into the air of sinuses as the skull undergoes significant accretion and remodeling, resulting in the loss of channels over time; (2) the screws may become embedded in the brain, which occurred both in swine that developed PTE and in those that did not develop PTE; and (3) the system does not allow remontage of the electrode array. Export of the EEG to other universal data file formats is necessary for advanced analysis.

Potential alterations to this system to address limitation disadvantages 1 and 2 could include using EEG subdural electrode strips spliced to the DSI implant using the DSI splice kit. These electrodes would slip underneath the dura. Disadvantage 3 could be addressed by installing two telemetry transponders in the neck/hemisphere allowing a montage with additional electrodes. With these improvements, skull screws would be avoided and *post hoc* remontaging and advanced signal analysis of the recording would be possible.

The methods described in this study demonstrate the feasibility of using swine to model post-traumatic epilepsy via video EEG using a commercially available radiotelemetry system. This system allowed for up to 13 months of monitoring producing good quality EEG. The setup was largely uncomplicated and required minimal upkeep of successfully implanted animals. This robust system may be of benefit to detect epilepsy in swine over the long period of epileptogenesis in this species. Slight modifications to this system as described may overcome the significant skull accretion in swine and improve the quality of EEGs acquired.

## Synthesis

Reviewing Editor: Katalin Toth, University of Ottawa Decisions are customarily a result of the Reviewing Editor and the peer reviewers coming together and discussing their recommendations until a consensus is reached. When revisions are invited, a fact-based synthesis statement explaining their decision and outlining what is needed to prepare a revision will be listed below. The following reviewer(s) agreed to reveal their identity: Richard Miles, Nora De Camp.

Both reviewers found the study interesting which will provide useful information for the research community. The manuscript should be strengthened on two fronts:

A) A more quantitative approach would significantly improve the study; details regarding this point are detailed in Rev.#1’s comments.

B) Please illustrate the stability of the quality of the recordings.

Rev.#1

This interesting study makes the case that post-traumatic epilepsies can best be studied in larger animals than rodents and cheaper animals than monkeys. The ms describes work in freely-moving pigs using commercially available material to record EEG signals over up to 14 mths in response to brain contusion. However the ms is more concerned with animal husbandry aspects of the work than with the neurobiology. If the authors wish to make the case that swine can feasibly be used to model post-traumatic epilepsy, they mist present the proportion of animals that did generate interictal and ictal-like events. The ictal records that are shown in one Figure might originate in just one out of 13 animals. Such a success rate would not encourage many other workers to use this model.

Major points

1, L132. Would be useful to define in greater detail the ‘5ms specificity’ between EEG and video achieved with the Network time protocol. What does it mean, how was it verified?

2, L196. Readers might like to know the mean, range and sd of weights for the pigs that were used.

3, L308. Would be useful to give the duration of a ‘typical’ surgery from induction of anesthesia until the start of recovery.

4, L373. Should define NPO, PO and IM somewhere.

5, L399. The ms needs to be more explicit on the appearance and timing of epileptiform activities. These data are needed to judge the success of a paper that describes a novel approach. Providing the data here will surely not impede publication of more detailed analysis in a subsequent paper.

The authors should present the latency to the emergence of interictal events, to the emergence of ictal events, the frequencies of both types of events in the weeks before recordings stopped and the number of animals in which they were detected.

6, L407. Fig.3 shows diverse forms of EEG events recorded from pigs that were treated with the cortical impact device. However EEG activity may not be epileptic. There is a large literature on the presence of physiological sharp waves in EEG records from rodents. Similar activity has been recorded in pigs (Ulyanova et al, 2019). The authors should either show data or add a sentence on the presence or absence of such events in EEG records from sham operated pigs.

7, L429. It is odd not to show any images in a ‘video-EEG’ study. Video observations seem to have been crucial in distinguishing movement artefacts (Fig. 4) and also in defining peri-ictal behavior. Showing one still image of both in this ms would not impair subsequent publication of more detailed analysis.

8, L456-476. The information in this paragraph on animal housing could be shortened and better placed in the methods section, close to the Animal husbandry procedures.

9, L478-502. Useful to give information on the perils of skull thickening over these long recordings. But details on electrode location are not sufficiently precise. All readers have is the schematic of Fig. 2C. How similar were electrode locations in the 13 test and 3 control pigs? Was a stereotaxic approach used, to fix the head and also to facilitate comparisons between animals and surgeries? Were similar gyri targeted in all animals? Did bio-potential leads rest on the cortical surface or were they inserted to some depth? More details should be given in the methods section.

10, L505-525. The material on treatment and prevention of infections could also be shortened and would be more appropriate in the methods section, with the Animal husbandry procedures.

11, 532-536. It is strange to discuss potential differences between delays to the emergence of epilepsy after brain trauma in rodents versus pigs without presenting any data from this work.

12, L595-596. The absence of quantitative data from this study means that solid conclusions cannot be reached on the feasibility of using swine to model post-traumatic epilepsy.

Minor points

1, L30. Biofidelic is not a very frequently used word. I had to look it up. Worse, it does not add much if anything to that sentence.

2, L181. ‘as space for additional space’ should be edited. Maybe the first two words aren’t needed.

3, L246. Instead ‘rongeurs’.

Rev.#2

A very well written manuscript, clear description of the techniques with only few questions left:

1. Electrode position looks different in figures 2b) and 2c)

2. term “injured” can be misunderstood, “lesioned” would be more explicit

3. Figure 5b: could you please add a dimension (cm?)
